# Assessments of Thioridazine as a Helper Compound to Dicloxacillin against Methicillin-Resistant *Staphylococcus aureus*: *In Vivo* Trials in a Mouse Peritonitis Model

**DOI:** 10.1371/journal.pone.0135571

**Published:** 2015-08-12

**Authors:** Michael Stenger, Kristoffer Hendel, Peter Bollen, Peter B. Licht, Hans Jørn Kolmos, Janne K. Klitgaard

**Affiliations:** 1 Research Unit of Clinical Microbiology, University of Southern Denmark, Odense, Denmark; 2 Department of Cardiothoracic Surgery, Odense University Hospital, Odense, Denmark; 3 Biomedical Laboratory, University of Southern Denmark, Odense, Denmark; 4 Department of Biochemistry and Molecular Biology, University of Southern Denmark, Odense, Denmark; Columbia University, UNITED STATES

## Abstract

**Introduction:**

The rise in antimicrobial resistance is a major global concern and requires new treatment strategies. The use of helper compounds, such as thioridazine (TDZ), an antipsychotic drug, in combination with traditional antibiotics must be investigated.

**Objectives:**

The aim of this study was to investigate the efficacy of TDZ as a helper compound for dicloxacillin (DCX) against methicillin-resistant *Staphylococcus aureus* (MRSA) *in vivo*, and compare the combination treatment of DCX+TDZ with vancomycin (VAN).

**Methods:**

Mice were inoculated with an intraperitoneal (IP) injection of MRSA (10^8^ CFU) and treated in a 12-hour cycle for 48 hours. By termination, bacterial quantities in a peritoneal flush, spleen and kidneys were obtained. In the main trial the drugs were administered subcutaneously in five treatment groups: 1) DCX, 2) TDZ, 3) DCX+TDZ, 4) VAN, 5) SALINE. Additional smaller studies with IP administration and higher subcutaneous dosages (×1.5 and ×4) of the drugs were subsequently performed.

**Results:**

In the main trial no significant differences were found between DCX+TDZ and DCX or TDZ alone (p≥0.121–0.999). VAN performed significantly better than DCX+TDZ on all bacteriological endpoints (p<0.001). Higher subcutaneous dosages of DCX and TDZ improved the antibacterial efficacy, but the combination treatment was still not significantly better than monotherapy. IP drug administration of DCX+TDZ revealed a significantly better antibacterial effect than DCX or TDZ alone (p<0.001) but not significantly different from VAN (p>0.999).

**Conclusion:**

In conclusion, TDZ did not prove to be a viable helper compound for dicloxacillin against MRSA in subcutaneous systemic treatment. However, IP-administration of DCX+TDZ, directly at the infection site resulted in a synergetic effect, with efficacy comparable to that of VAN.

## Introduction

The rise in antimicrobial resistance is a major global concern and classified as the third largest threat to human health by the World Health Organisation (WHO) [1]. Methicillin-resistant *Staphylococcus aureus* (MRSA) is of special concern as it is widespread and often multi-resistant, thus hard to treat and related to poor patient outcome [2]. Previously, MRSA was primarily isolated in hospital settings, but community-associated MRSA and strains from livestock have emerged and increased the accumulated burden [3].

During the past 40 years, only two new antibiotic classes (daptomycin and linezolid) for the treatment of MRSA have been discovered and marketed and historically resistant strains have emerged to each new antibiotic introduced [4]. Consequently, there is an urgent need for novel ideas to manage MRSA.

A novel strategy is to use helper compounds in combination with traditional antibiotics. Helper compounds are drugs approved for other purposes, which also have varying degrees of antibacterial activity. Thioridazine (TDZ), an antipsychotic drug, is an example of such a helper compound with promising potential. Several *in vitro* studies have demonstrated that TDZ can re-sensitize MRSA [5–8] and significantly increase the sensitivity of methicillin-sensitive *Staphylococcus aureus* (MSSA) to *β*-lactam antibiotics [9]. Thus, the potentiated efficacy of TDZ and a *β*-lactam antibiotic in combination against *S*. *aureus* is referred to as a synergetic effect.

The antibacterial mechanisms of TDZ have previously only been linked to inhibition of efflux-pumps [10, 11], but recent studies have shown that TDZ induces major changes in gene expression in pathways such as cell wall biosynthesis, including penicillin-binding proteins (PBPs) [5, 12].

Despite these promising *in vitro* results, only two animal studies have recently been published on this specific drug combination against *S*. *aureus* with conflicting results [13, 14].

We have set up a modified mouse peritonitis model to test the *in vivo* viability of the combination treatment of TDZ and dicloxacillin (DCX) against MRSA. Additionally, the combination treatment is compared to the current clinical gold standard treatment against MRSA, vancomycin (VAN).

## Materials and Methods

### Antimicrobial agents and dosages

Dicloxacillin (Diclocil, Bristol-Myers Squibb) and Vancomycin (Vancomycin, Fresenius Kabi, Denmark) were purchased and used as the commercial product registered in Denmark for parenteral clinical use. Thioridazine (Thioridazine hydrochloride, Sigma-Aldrich Corporation, Denmark) was purchased and used in its racemic form.

Dosages were set on behalf of the given references in Table 1 and considerations on clinical applicability in humans. However, VAN was intentionally and according to the study by Docobo-Perez et al. [15] set at a high dose compared to the equivalent dose in humans. The rationale was to make sure that vancomycin had been administered in adequate dosages in order to be a useful positive control, and to minimize the risk of a type II error when comparing other treatments to vancomycin.

**Table 1:** Antimicrobial agents and dosages in the main trial.

Antimicrobial agent	Dose (Main trial)	Equivalent dose in mice (25 g)	Equivalent dose in humans (70 kg)
Dicloxacillin (DCX)	60 mg/kg/day [16]	1.5 mg/day	4.2 g/day
Thioridazine (TDZ)	4 mg/kg/day [17, 18]	0.1 mg/day	280 mg/day
Vancomycin (VAN)	220 mg/kg/day [15, 19]	5.5 mg/day	15.4 g/day

Antimicrobial agents and dosages utilized in the main trial. Equivalent dosages in mice and humans are listed.

According to the equivalent daily dosages in mice (Table 1), the antimicrobial agents were dissolved in isotonic saline (Amgros I/S, Denmark) at concentrations fitted for an injection of 0.5 ml twice a day. Mice treated with the combination treatment (DCX +TDZ) had two injections at different sites to avoid the possibility of crystallization or altered absorption when the drugs were mixed. Hence, this group of mice had a total volume of 1 ml twice a day. TDZ was at all times before injection protected from sunlight due to its decomposing effect on the drug.

### Bacterial strain, MIC and viability assay

We used a MRSA variant (XEN 31—Caliper LifeSciences) derived from MRSA ATCC 33591. The ATCC 33591 strain was previously used and validated as a virulent strain in other reference studies [5, 6, 14]. This MRSA variant also had bioluminescent properties, but this capacity was not utilized in the present study. MIC values were tested for all trial drugs by macro broth dilutions according to the principles described by Wiegand et al. [20], DCX: 32 mg/L; TDZ: 32 mg/L; VAN: 2 mg/L. *In vitro* synergy was confirmed by growth and viability assays as previously described by Klitgaard et al. [6] (data not shown).

### Animals and experimental conditions

Outbred albino female NMRI mice (NMRI-F, Taconic Denmark) with a mean starting weight of 28.4 (SD: 2.4) grams were used. They were kept 4–8 mice per cage with unrestricted access to food and water. After one week of acclimatization, a temperature transponder unit (BMDS) was injected subcutaneously during short inhalation anaesthesia with Isofluran “Baxter”. Afterwards, the mice were moved to a biosafety level II facility and given another two days of recovery before inoculation.

### Mouse peritonitis model

We used a previously described and widely used mouse peritonitis model for measuring antibiotic effect *in vivo* [21, 22], which was further modified by a LD0 threshold inoculum calibration and an extended treatment period to allow for evaluation of treatment efficacy by quantitative bacteriological endpoints and to make the model as clinically applicable as possible. The protocol was approved by the Danish Animal Experimentation Inspectorate (license no. 2013-15-2934-00866).

All mice were inoculated with an intraperitoneal (IP) injection of 10^8^ CFU of MRSA in 0.5 ml isotonic saline solution. This inoculum was calibrated as the highest tested concentration at which untreated mice showed clear signs of illness, but none had to be sacrificed according to the humane endpoints. We named this inoculum “LD0 threshold” since we noted that only a 2-fold increase in bacterial concentration (2×10^8^ CFU) caused approximately 60% of the mice to meet terminal humane endpoints, and thus would be sacrificed within the first 12 hours. The trial period was set to 48 hours based on pilot studies, which showed that untreated mice declined in weight during the first 48 hours and then recovered over the next couple of days, indicating spontaneous remission (data not shown).

Treatment was initiated two hours after inoculation and repeated every 12 hours. During the entire trial period, the mice were given analgesic treatment with buprenorphin (Temgesic) 0.05 mg/kg every 8 hours [23]. After 48 hours of treatment, the mice were sacrificed by swift cervical dislocation and subjected to a peritoneal flush (p-flush) with 2 ml isotonic saline. Following a short massage of the abdomen, >0.5 ml of the fluid was extracted and the spleen and kidneys were harvested. Spleen and kidneys were weighed and homogenised in 10 ml sterile phosphate buffered saline (PBS—Statens Serum Institut, Denmark). All three solutions from p-flush, spleen and kidneys were individually plated out on Mueller-Hinton (MH) agar plates in ten-fold dilutions with 100 μL on each plate. The plates were incubated at 37°C for 24 hours and colonies were counted.

### Trials

The main trial was conducted in a total of 136 mice divided into five treatment groups (n): DCX (28), TDZ (28), DCX+TDZ (28), VAN (28), and SALINE (24). The drugs were administered subcutaneously as described above. Subsequently, additional experiments were carried out in a smaller number of mice to investigate the outcome of IP administration and higher subcutaneous dosages of the drugs. To compensate for the smaller number of mice in these additional trials, all three bacteriological endpoints for each mouse were pooled into a total CFU/mL value.

### Humane endpoints

Weight and temperature was measured, and behaviour and appearance was observed and scored for each individual animal before inoculation (baseline), every 12 hours during the trial period and before sacrifice. Assessments of behaviour and appearance were based on a number of specific parameters, such as level of activity, eye conditions, abnormal behaviour, diarrhea, change in fur, and hunchback (S2A and S2B Table). Monitoring humane endpoints in infectious models with respect to animal health, well-being and safety is mandatory by the Danish Animal Experimentation Inspectorate. Furthermore, data on humane endpoints (especially weight) may also complement the bacteriological endpoints as an indirect marker for treatment efficacy [24].

### Statistical analyses

Bacterial count data for all three primary bacteriological endpoints (p-flush, spleen and kidneys) were converted into CFU/mL and transformed by natural logarithm (ln) to obtain a normal distribution, which were visually and statistically confirmed by inverse normal plots and Shapiro-Wilk tests, hence displayed as ln(CFU/mL). Bacterial counts for spleen and kidneys were also corrected relative to the mean weight of the organs in the related treatment group ((organ weight / mean organ weight in the related treatment group) x bacterial count). Furthermore, a fourth bacteriological endpoint (total) was generated by pooling the three primary bacteriological endpoints.

Weight data were normalized and displayed as a percentage, with baseline weight being 100%.

One-way ANOVA followed by the conservative Bonferroni multiple comparisons test adjusting P-value for each comparison was used. Simple linear regression and Pearson’s correlation coefficient were used to describe the association between weight and total CFU/mL for all three bacteriological endpoints in the main trial. Box-whisker plots are presented as median values, together with 25 and 75 percentiles, as well as minimum and maximum values. A p-value of <0.05 was considered significant. Stata/IC version 13.1 (Statacorp) was used for illustrations and analysis.

## Results

### Main trial

**Fig 1:**
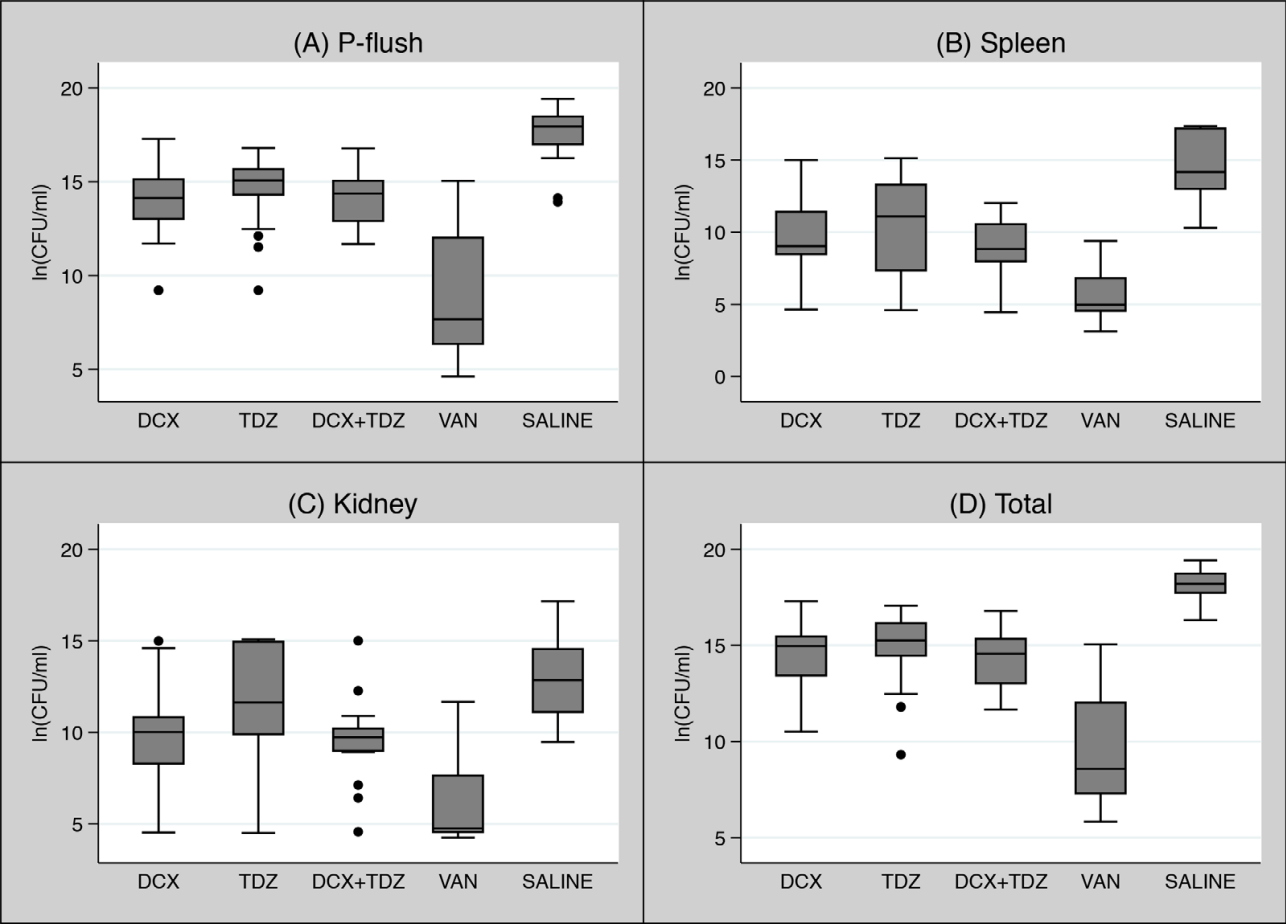
A-D: Main trial: Box-whisker plots of bacterial quantities displayed as ln(CFU/mL) in all treatments groups sorted by bacteriological endpoints–(A) P-flush, (B) Spleen, (C) Kidney, and (D) Total. The filled dots indicate outliers. DCX: Dicloxacillin; TDZ: Thioridazine; VAN: Vancomycin; SALINE: Isotonic saline.

As presented in Fig 1A–1D, mice treated with the negative control, isotonic saline (SALINE), had the highest quantity of bacteria in all bacteriological endpoints compared to all other treatment groups (p<0.001), except one comparison between TDZ and SALINE in the kidney endpoint (p = 0.597) (Fig 1C). An overall consistent reduction in the amount of bacteria was found in mice treated with the positive control VAN.

We found no statistically significant differences in bacterial quantities between DXC+TDZ and DCX or TDZ alone in p-flush (p>0.999) (Fig 1A), spleen (p>0.999 and p = 0.578, respectively) (Fig 1B), kidney (p>0.999 and p = 0.121, respectively) (Fig 1C), and total (p>0.999) (Fig 1D). However, the quantities of bacteria in the VAN group were significantly lower in all bacteriological endpoints compared to the DCX, TDZ, and DCX+TDZ (p<0.001) (Fig 1A–1D and S1 Fig).

### Correlation between bacterial quantity and weight

**Fig 2:**
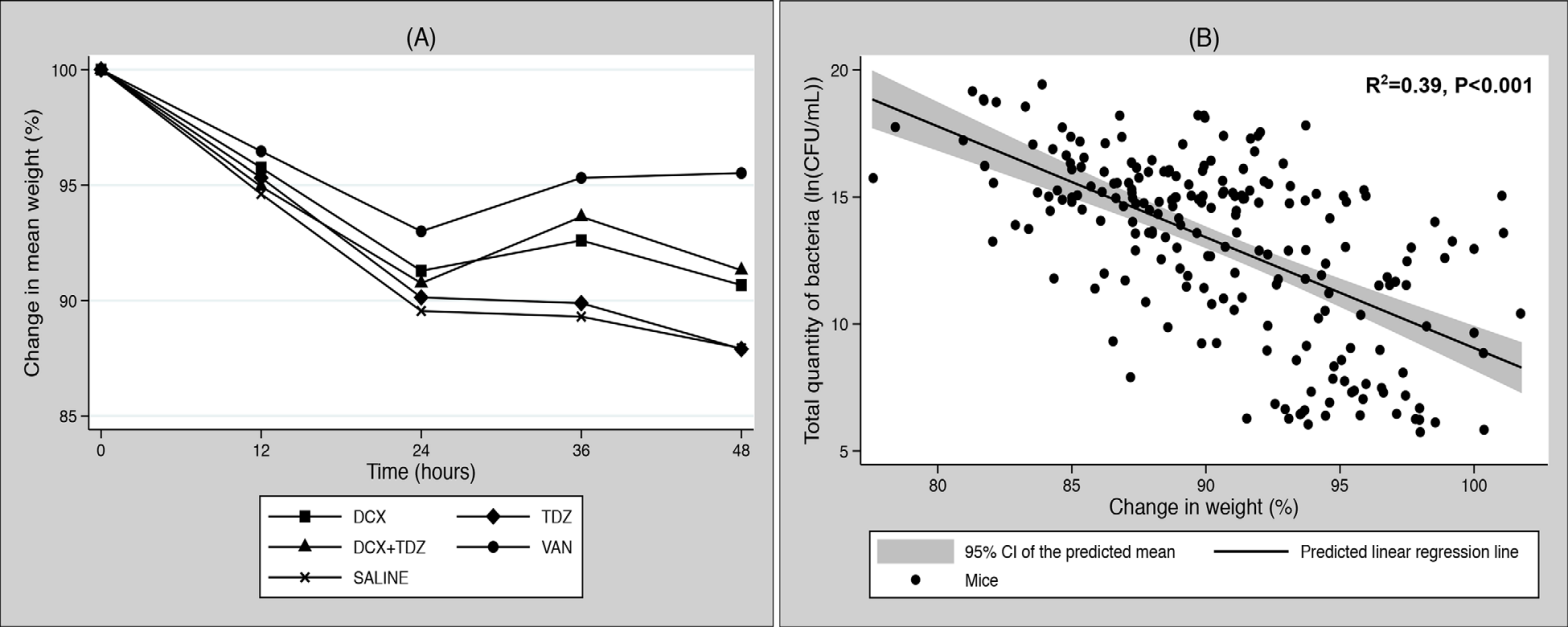
A + B: Main trial: (A) Development in mean weight displayed as a percentage of baseline weight (100%) within each treatment group during the 48-hour trial period. DCX: Dicloxacillin; TDZ: Thioridazine; VAN: Vancomycin; SALINE: Isotonic saline. **(B)** Scatter plot with a predicted linear regression line and a 95% CI (grey zone) of the predicted mean showing the correlation between the total quantity of bacteria and total change in weight during the entire trial period displayed as a percentage of baseline weight (100%).

Changes in mean weight within each treatment group during the trial period are shown in Fig 2A. We observed an initial weight loss for all treatment groups during the first 24 hours. Over the following 24 hours, VAN-treated mice recovered some of their weight loss, whereas the weight of DCX+TDZ- and DCX-treated mice did not recover. Finally, the weight of mice treated with TDZ or SALINE continued to decline.

The linear regression analysis between the total amount of bacteria and changes in weight proved highly significant (p<0.001, R^2^ = 0.39) and with a correlation coefficient of -0.62. A scatterplot with a fitted line including a 95% confidence intervals (CI) of the predicted mean is shown in Fig 2B.

Anova analysis on changes in weight showed significantly less weight loss in the VAN group compared to all other treatment groups (p≤0.001) but no statistically significant differences between DCX+TDZ, DCX,TDZ, or SALINE (p≥0.069–0.999).

### IP trial

Forty-eight mice in total (12 mice per group) were given the same dosages as used in the main trial, but by IP administration of the drugs instead of subcutaneously.

**Fig 3:**
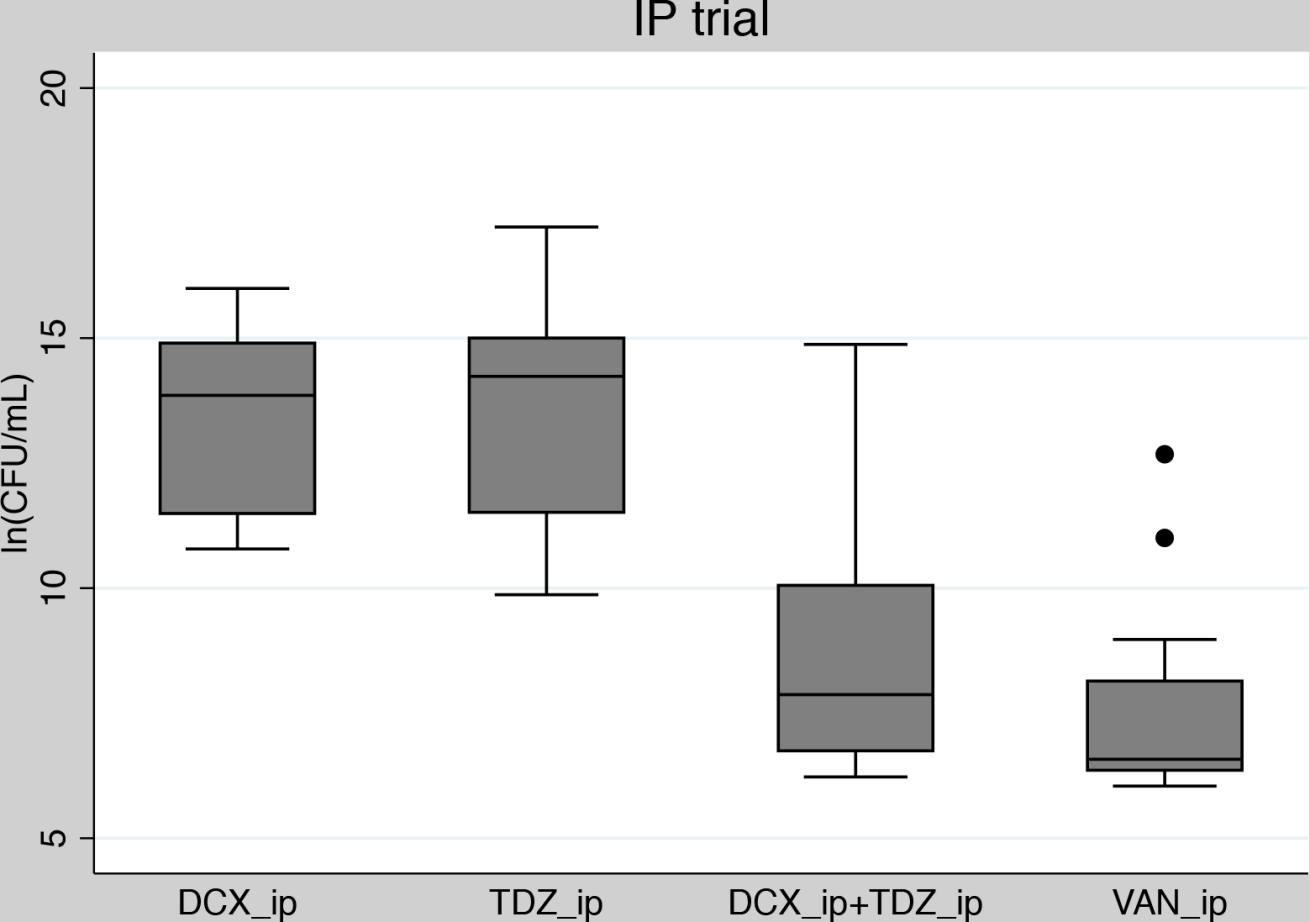
IP trial: Box-whisker plot of pooled bacteriological endpoints related to treatment groups in the IP-trial. The filled dots indicate outliers. DCX_ip: Dicloxacillin administered IP; TDZ_ip: Thioridazine administered IP; VAN_ip: Vancomycin administered IP.

As presented in Fig 3, mice treated with the IP combination treatment (DCX_ip+TDZ_ip) had a significantly lower quantity of bacteria than mice treated with DCX_ip or TDZ_ip in monotherapy (p<0.001). Furthermore, the reduction in bacterial quantity seen in DCX_ip+TDZ_ip was statistically comparable to that seen in VAN_ip (p>0.999) (S2 Fig).

### High dose trials

In a total of 56 mice (8 mice per group) we ran trials with increased subcutaneous dosages for DCX and TDZ of ×1.5 and ×4 compared to the main trail. All groups were compared against the VAN group from the main trial.

**Fig 4:**
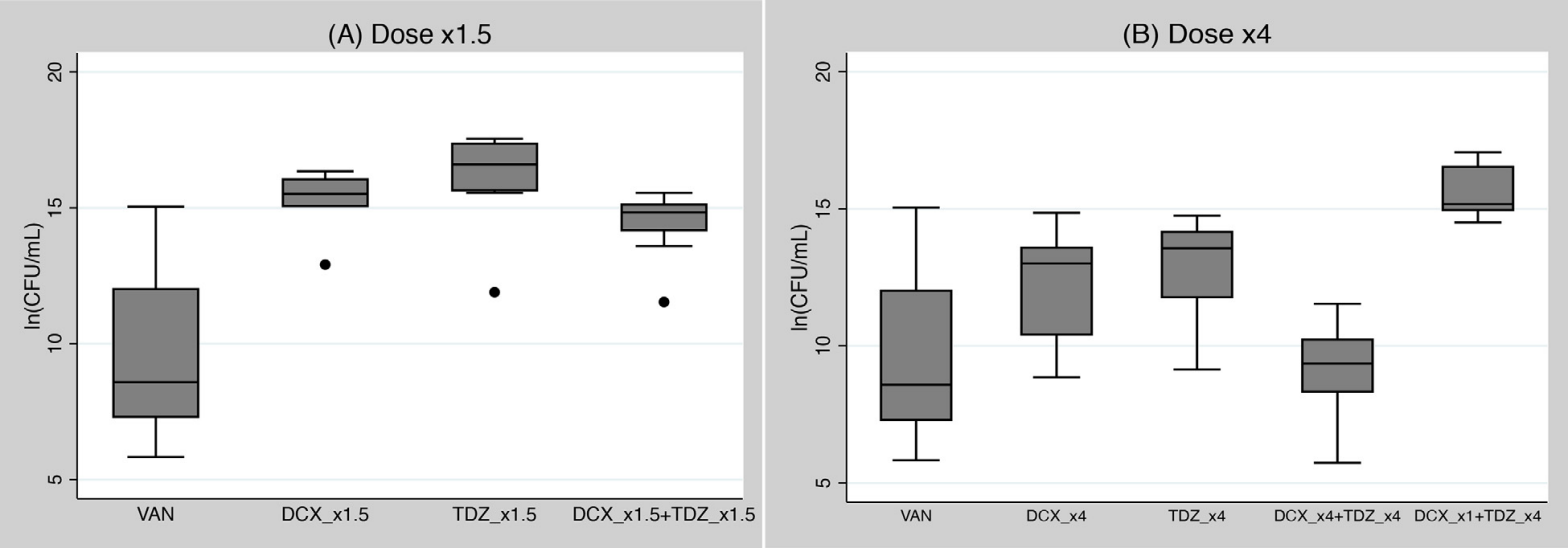
A + B: High dose trials: Box-whisker plots of pooled bacteriological endpoints related to treatment groups in the two high dose trials, (A) Dose ×1.5 and (B) Dose ×4. The filled dots indicate outliers. DCX: Dicloxacillin; TDZ: Thioridazine; VAN: Vancomycin (same dose as in the main trial); _x1: same dose as in the main trial _x1.5: One and a half times higher dose than in the main trail; _x4: Four times higher dose than in the main trail.

No statistically significant differences were found in the quantities of bacteria between DCX_x1.5+TDZ_x1.5 and monotherapy of TDZ_x1.5 or DCX_x1.5 (p>0.999). All ×1.5 dose groups had statistically higher amounts of bacteria than the VAN group from the main trial (p<0.001) (Fig 4A).

The bacterial quantities were considerably reduced in the dose x4 trial, but still without statistically significant differences between the combination of DCX_x4+TDX_x4 and DCX_x4 or TDZ_x4 alone (p = 0.143 and p = 0.057, respectively) (Fig 4B). The combination of DCX_x4+TDX_x4 and DCX_x4 alone performed equally as well as the VAN group from the main trial (p>0.999 and p = 0.122, respectively). TDZ_x4 tested marginally inferior to VAN (p = 0.039). Finally, the combination group with mixed dosages (DCX_×1+TDZ_×4) had a significant higher quantity of bacteria than VAN and DCX_×4+TDZ_×4 (p<0.001) but not statistically different from DCX_×4 or TDZ_×4 (p = 0.169 and p = 0.379, respectively) (S2 Fig).

Anecdotally, we observed that all mice treated with TDZ were more docile and restful than other mice, and (especially in the dose x4 trial) were considerably more prone to inactivity, abnormal behaviour, major changes in fur, and bleeding from injecting sites.

## Discussion

Several *in vitro* studies have demonstrated the synergetic effect of combining TDZ and β-lactams against MRSA [5–8], but these results have just recently been reproduced in a multicellular worm model. *C*. *elegans* were exposed to MRSA over a three-day period and subsequently treated with TDZ (8 mg/mL) and DCX (8 mg/mL) in combination or alone for further two days. The combination treatment induced a 14-fold reduction in bacterial load in contrast to a 2 or 3-fold reduction by each drug alone compared to untreated animals [14]. In contrast, another recent study found no synergetic effect of TDZ and a β-lactam antibiotic (cefazolin) against MRSA in a mouse skin infection model [13]. In this animal model, TDZ was administrered IP one hour prior to the cutaneous inoculation of the skin and subsequently once per day in a dose of 3–30 mg/kg. Cefazolin was administrered subcutaneously in doses of 25–50 mg/kg following the inoculation and once per day onwards.

Interestingly, the results from the present study concur with these conflicting *in vivo* studies. Similar to Poulsen et al. [14] we found a significant reduction in total bacterial load and a synergetic effect when the combination treatment was administered IP directly at the infection site. Furthermore, the combination treatment performed equally as well as VAN_ip, although, VAN was purposely set to a high dose for a strong comparison. We emphasize that our results from the IP trial were obtained in a relatively small number of mice, and these two simple experiments roughly resemble *in vitro* conditions by providing direct contact between bacteria and drugs. However, when the combination treatment of DCX and TDZ is administered systemically (away from the infection site) the model becomes more complex.

In line with Hahn et al. [13], we found no synergetic effect against MRSA in the present mouse peritonitis model when the drugs were administered subcutaneously or away from the infection site. Furthermore, the combination treatment revealed to be significantly less effective in comparison to VAN in all bacteriological endpoints, although this finding could be related to the purposely high VAN dose utilized. Consequently, we investigated the possibility of having utilized inadequate subcutaneous dosages of DCX and/or TDZ. No trends towards synergy or increased antibacterial efficacy were found in the dose x1.5 trial. However, in the dose x4 trial a trend towards a synergetic effect was observed, although the results were not statistically significant. Furthermore, we noted an increased antibacterial efficacy of the combination treatment (DCX_x4+TDZ_x4) and DCX_x4 alone, which were statistically equal to the efficacy of VAN from the main trial. This may indicate that MIC concentrations or higher were reached for DCX, and thus limited the effect of adding TDZ. Consequently, we tested the combination treatment with mixed dosages (DCX_x1+TDZ_x4), which gave similar results as the monotherapy, but not as effective as VAN. Collectively, the results from the high dose trials might be limited by small sample sizes, but still no clear tendency towards a synergetic effect was observed as reported in the IP trial. Additionally, we observed a substantial rise in adverse effects, such as inactivity, abnormal behaviour, major changes in fur, and bleeding from injecting sites with increasing TDZ doses.

We speculate that the lack of antibacterial efficacy by subcutaneous treatment might be related to high protein binding of TDZ, and thus limited drug penetration into the peritoneal fluid [25]. TDZ may have the ability to accumulate in macrophages by lysosomal trapping to a level 10–300 fold above the concentration in the medium or plasma [26] and *ex vivo* studies on human macrophages have proven the feasibility of killing phagocytosed MRSA at clinically relevant concentrations of TDZ [27], but so far this has not been confirmed *in vivo* by animal or human studies. Furthermore, the two metabolites mesoridazine and sulforidazine are considered to be more potent with regards to antipsychotic capacity, but how well this translates to the antimicrobial effect is still unexplored [28].

TDZ including other phenothiazines and thioxanthenes have been tested in other mouse models against different bacterial strains with positive results, but these studies are also limited to simple setups with primed (pre-infection) and/or localized treatment [17, 29–31].

The present mouse peritonitis model was intended to be as clinically relevant as possible with no pre-inoculation treatment and drug administration twice daily, although in a clinical setting DCX is usually administered three times daily. We chose not to apply this regimen to our design because TDZ and VAN is ideally administered twice daily. Overall, this would have required different drug administration regimens among different treatment groups, which could bias the results. The carefully calibrated LD0 threshold inoculum provided a reproducible quantative model with evalution of multiple bacteriological endpoints instead of classic survival studies with binary outcomes. Although this approach may be limited by a fairly high spread in bacterial quantities within the same treatment group (S1 Table), the validity was confirmed by consistently high quantities of bacteria in all three primary bacteriological endpoints in mice treated with the negative control (SALINE) and similar low bacterial quantities in the positive control (VAN). Additionally, the robustness of the results from the main trial was confirmed by the same findings when we used changes in weight as an indirect endpoint. The widely accepted assumption of weight being a valuble indicator of animal well-being and thereby treatment efficacy in infectious animal models was confirmed by a highly significant regression analysis and a good corellation to total bacterial load. Finally, the LD0 threshold design accompanied by humane endpoints is an example of refinement in modern infectious animal research with a substantial improvement in animal welfare without compromising research results.

In conclusion: in this present *in vivo* mouse peritonitis model, TDZ did not prove to be a viable helper compound to DCX against MRSA when the drug combination was administered systemically with repeated subcutaneous injections. The antibacterial efficacy of TDZ alone or in combination seemed to increase with higher utilized dosages but so did the observed adverse effects, and still no significant synergetic effect was observed. However, when the combination treatment was administered IP directly at the infection site, a highly significant synergetic effect comparable with VAN was found. This is in full accordance with results obtained in previous animal studies testing topical treatment with combinations of TDZ and *β*-lactam antibiotics against *S*. *aureus* [13, 14]. Hence, despite the to limitations discussed above, TDZ may become a useful helper compound to β-lactams in localized treatment of MRSA. We call for larger studies to confirm this important observation, and to clarify why systemic treatment with the drug combination appeared ineffective.

## Supporting Information

S1 Fig Checkerboards on ANOVA analysis in the main trial. Main trial: ANOVA analysis of bacterial quantities related to treatment groups sorted by bacteriological endpoints—(A) P-flush, (B) Spleen, (C) Kidney, and (D) Total. DCX: Dicloxacillin; TDZ: Thioridazine; VAN: Vancomycin; SALINE: Isotonic saline; (n) number of mice included in each treatment group.
(DOCX)

S2 Fig Checkerboards on ANOVA analysis in the additional trials. ANOVA analysis of total bacterial load (pooled p-flush, spleen and kidneys) related to treatment groups. IP trial: Intraperitoneal administration of the drugs. Dose x1.5/x4: Subcutaneous administration of 1.5/4 times higher dosages than in the main trial. DCX: Dicloxacillin; TDZ: Thioridazine; VAN: Vancomycin; SALINE: Isotonic saline; (n) number of mice included in each treatment group.
(DOCX)

S1 Table Mean values of bacterial quantities for all three primary bacteriological endpoints sorted by all treatment groups. (DOCX)

S2 Table Assessment of behaviour and appearance. **(A)** Evaluation of behaviour and appearance was done on different parameter: Level of activity, eye conditions, abnormal behaviour, diarrhea, hunchback, and change in fur, by which each animal acquired a total score (sum of points). **(B)** Interpretation of the total score for each animal on the level of stress and the related actions taken.
(DOCX)

S3 Table Baseline values of mean weight and temperature sorted by all treatment groups. (DOCX)

S1 Text ARRIVE Checklist. (PDF)
