# Supplementary material for: Assessments of Thioridazine as a Helper Compound to Dicloxacillin against Methicillin-Resistant Staphylococcus aureus: In Vivo Trials in a Mouse Peritonitis Model
Source: PLoS One. 2015 Aug 12;10(8):e0135571. doi: 10.1371/journal.pone.0135571 (PMC4534400; doi:10.1371/journal.pone.0135571)
Supplement: S1 Fig — Main trial: ANOVA analysis of bacterial quantities related to treatment groups sorted by bacteriological endpoints—(A) P-flush, (B) Spleen, (C) Kidney, and (D) Total. DCX: Dicloxacillin; TDZ: Thioridazine; VAN: Vancomycin; SALINE: Isotonic saline; (n) number of mice included in each treatment group. (DOCX) [file pone.0135571.s001.docx]

**S1 Fig. Checkerboards on ANOVA analysis in the main trial**

| **Main trial:** *(A) P-flush* | | | | |
| --- | --- | --- | --- | --- |
| vs | DCX |  |  |  |
| TDZ | > 0.999 | TDZ |  |  |
| DCX+TDZ | >0.999 | >0.999 | DCX+TDZ |  |
| VAN | <0.001 | <0.001 | <0.001 | VAN |
| SALINE | <0.001 | <0.001 | <0.001 | <0.001 |
| Treatment group (n): DCX (25), TDZ (25), DCX+TDZ (22), VAN (27), SALINE (22) | | | | |
|  |  |  |  |  |
|  |  |  |  |  |
| **Main trial:** *(B) Spleen* | | | | |
| vs | DCX |  |  |  |
| TDZ | >0.999 | TDZ |  |  |
| DCX+TDZ | >0.999 | 0.578 | DCX+TDZ |  |
| VAN | <0.001 | <0.001 | <0.001 | VAN |
| SALINE | <0.001 | <0.001 | <0.001 | <0.001 |
| Treatment group (n): DCX (26), TDZ (26), DCX+TDZ (20), VAN (27), SALINE (18) | | | | |
|  |  |  |  |  |
|  |  |  |  |  |
| **Main trial:** *(C) Kidney* | | | | |
| vs | DCX |  |  |  |
| TDZ | 0.143 | TDZ |  |  |
| DCX+TDZ | >0.999 | 0.121 | DCX+TDZ |  |
| VAN | <0.001 | <0.001 | <0.001 | VAN |
| SALINE | <0.001 | 0.597 | 0.001 | <0.001 |
| Treatment group (n): DCX (24), TDZ (26), DCX+TDZ (20), VAN (28), SALINE (21) | | | | |

| **Main trial:** *(D) Total* | | | | |
| --- | --- | --- | --- | --- |
| vs | DCX |  |  |  |
| TDZ | >0.999 | TDZ |  |  |
| DCX+TDZ | >0.999 | >0.999 | DCX+TDZ |  |
| VAN | <0.0001 | <0.0001 | <0.0001 | VAN |
| SALINE | <0.0001 | <0.0001 | <0.0001 | < 0.0001 |
| Treatment group (n): DCX (24), TDZ (25), DCX+TDZ (20), VAN (27), SALINE (17) | | | | |
