# Supplementary material for: Assessments of Thioridazine as a Helper Compound to Dicloxacillin against Methicillin-Resistant Staphylococcus aureus: In Vivo Trials in a Mouse Peritonitis Model
Source: PLoS One. 2015 Aug 12;10(8):e0135571. doi: 10.1371/journal.pone.0135571 (PMC4534400; doi:10.1371/journal.pone.0135571)
Supplement: S2 Fig — ANOVA analysis of total bacterial load (pooled p-flush, spleen and kidneys) related to treatment groups. IP trial: Intraperitoneal administration of the drugs. Dose x1.5/x4: Subcutaneous administration of 1.5/4 times higher dosages than in the main trial. DCX: Dicloxacillin; TDZ: Thioridazine; VAN: Vancomycin; SALINE: Isotonic saline; (n) number of mice included in each treatment group. (DOCX) [file pone.0135571.s002.docx]

**S2 Fig. Checkerboards on ANOVA analysis in the additional trials**

| **IP trial** | | | |
| --- | --- | --- | --- |
| vs | DCX_ip |  |  |
| TDZ_ip | >0.999 | TDZ_ip |  |
| DCX_ip+TDZ_ip | <0.001 | <0.001 | DCX_ip+TDZ_ip |
| VAN_ip | <0.001 | <0.001 | >0.999 |
| Treatment group (n): DCX_ip (12), TDZ_ip (12), DCX_ip+TDZ_ip (12), VAN_ip (12) | | | |

| **Dose x1.5** | | | |  |
| --- | --- | --- | --- | --- |
| vs | VAN |  |  |  |
| DCX_x1.5 | <0.001 | DCX_x1.5 |  |  |
| TDZ_x1.5 | <0.001 | >0.999 | TDZ_x1.5 |  |
| DCX_x1.5+TDZ_x1.5 | <0.001 | >0.999 |  |  |
| Treatment group(n): DCX_x1.5(7), TDZ_x1.5 (8), DCX_x1.5+TDZ_x1.5 (8), VAN(27) | | | |  |
|  |  |  |  |  |
| **Dose x4** | | | | |
| vs | VAN |  |  |  |
| DCX_x4 | 0.122 | DCX_x4 |  |  |
| TDZ_x4 | 0.039 | >0.999 | TDZ _x4 |  |
| DXC_x4+TDZ_x4 | >0.999 | 0.143 | 0.057 | DXC_x4+TDZ_x4 |
| DCX+TDZ_x4 | <0.001 | 0.169 | 0.379 | <0.001 |
| Treatment group (n): DCX_x4 (7), TDZ_x4 (7), DCX_x4+TDZ_x4 (8), DCX+TDZ_x4 (7), VAN (27) | | | | |
