# Supplementary material for: Assessments of Thioridazine as a Helper Compound to Dicloxacillin against Methicillin-Resistant Staphylococcus aureus: In Vivo Trials in a Mouse Peritonitis Model
Source: PLoS One. 2015 Aug 12;10(8):e0135571. doi: 10.1371/journal.pone.0135571 (PMC4534400; doi:10.1371/journal.pone.0135571)
Supplement: S1 Table — (DOCX) [file pone.0135571.s003.docx]

**S1 Table. Mean values of bacterial quantities for all three primary bacteriological endpoints sorted by all treatment groups**

| **Treatment group** | **Mean values** (ln(CFU/ml) (95% CI)) | | |
| --- | --- | --- | --- |
|  | **P-flush** | **Spleen** | **Kidney** |
| **DCX** | 13.9 (13.4-14.8) | 9.7 (8.6-10.9) | 9.7 (8.7-10.7) |
| **TDZ** | 14.5 (13.8-15.2) | 10.4 (9.0-11.8) | 11.4 (10.3-12.8) |
| **DCX+TDZ** | 14.1 (13.5-14.8) | 9.0 (8.2-9.8) | 9.6 (8.6-10.5) |
| **VAN** | 8.9 (7.8-10.4) | 5.6 (4.9-6.0) | 6.2 (5.1-6.8) |
| **SALINE** | 17.5 (17.1-18.3) | 14.4 (13.1-15.4) | 12.8 (11.8-13.7) |
| **DCX_ip** | 12.4 (10.7-14.1) | 8.7 (7.1-10.3) | 9.0 (7.6-10.3) |
| **TDZ_ip** | 10.0 (7.8-12.3) | 7.5 (6.3-8.7) | 10.8 (8.5-13.1) |
| **DCX_ip+TDZ_ip** | 7.4 (5.7-9.1) | 5.7 (4.7-6.7) | 6.8 (5.2-8.4) |
| **VAN_ip** | 6.5 (5.2-7.7) | 5.6 (4.6-6.6) | 4.7 (4.5-5.0) |
| **DCX_x1.5** | 13.5 (10.5-16.6) | 8.9 (5.7-12.0) | 11.9 (10.0-13.8) |
| **TDZ_x1.5** | 15.1 (13.3-16.8) | 13.0 (10.4-15.5) | 13.3 (12.4-14.2) |
| **DCX_x.1.5+TDZ_x1.5** | 14.1 (13.3-14.9) | 9.7 (6.8-12.6) | 10.0 (7.5-12.4) |
| **DCX_x4** | 8.9 (6.4-11.5) | 4.5 (4.3-4.7) | 11.1 (8.4-13.7) |
| **TDZ_x4** | 10.1 (7.2-13.0) | 4.5 (4.4-4.6) | 10.7 (7.1-13.4) |
| **DCX_x4+TDZ_x4** | 8.8 (7.3-10.2) | 5.4 (4.3-6.5) | 5.7 (4.6-6.8) |
| **DCX_x1+TDZ_x4** | 15.1 (14.2-16.0) | 12.5 (10.8-14.3) | 9.9 (7.9-11.9) |
