# Supplementary material for: Assessments of Thioridazine as a Helper Compound to Dicloxacillin against Methicillin-Resistant Staphylococcus aureus: In Vivo Trials in a Mouse Peritonitis Model
Source: PLoS One. 2015 Aug 12;10(8):e0135571. doi: 10.1371/journal.pone.0135571 (PMC4534400; doi:10.1371/journal.pone.0135571)
Supplement: S2 Table — (A) Evaluation of behaviour and appearance was done on different parameter: Level of activity, eye conditions, abnormal behaviour, diarrhea, hunchback, and change in fur, by which each animal acquired a total score (sum of points). (B) Interpretation of the total score for each animal on the level of stress and the related actions taken. (DOCX) [file pone.0135571.s004.docx]

**S2 Table. Assessment of behaviour and appearance**

**S2A Table:**

| **Level of activity** |  | **Score (points)** |
| --- | --- | --- |
| Normal |  | 0 |
| Low |  | 1 |
| No |  | 2 |
|  |  |  |
| **Eye conditions** |  |  |
| No affection |  | 0 |
| Mild affection |  | 1 |
| One eye closed |  | 2 |
| Both eyes closed |  | 3 |
|  |  |  |
| **Miscellaneous** |  |  |
| Abnormal behaviour |  | 1 |
| Diarrhea |  | 1 |
| Hunchback |  | 1 |
| Changes in fur |  | 1 |

**S2B Table:**

| **Total score (points):** |  | **Level of stress** |  | **Actions** |
| --- | --- | --- | --- | --- |
| 0 |  | No stress |  | No action |
| 1-4 |  | Mild stress |  | No action |
| 5-6 |  | Moderate stress |  | Increased control/follow-up |
| 7 |  | Severe stress |  | Additional analgesia + assessment by veterinarian |
| 8-9 |  | Extreme stress |  | Sacrifice the animal |
