# Supplementary material for: Assessments of Thioridazine as a Helper Compound to Dicloxacillin against Methicillin-Resistant Staphylococcus aureus: In Vivo Trials in a Mouse Peritonitis Model
Source: PLoS One. 2015 Aug 12;10(8):e0135571. doi: 10.1371/journal.pone.0135571 (PMC4534400; doi:10.1371/journal.pone.0135571)
Supplement: S3 Table — (DOCX) [file pone.0135571.s005.docx]

**S3 Table. Baseline values of mean weight and temperature sorted by all treatment groups**

| **Treatment group** | **Baseline values** | |
| --- | --- | --- |
|  | **Mean weight (g) (SD)** | **Mean temperature (°C) (SD)** |
| **DCX** | **28.8 (2.3)** | **37.6 (0.9)** |
| **TDZ** | **28.9 (3.0)** | **37.3 (0.8)** |
| **DCX+TDZ** | **29.3 (3.6)** | **37.2 (0.8)** |
| **VAN** | **28.7 (2.4)** | **37.3 (0.9)** |
| **SALINE** | **27.0 (1.1)** | **37.8 (0.4)** |
| **DCX_ip** | **29.3 (1.2)** | **36.7 (0.5)** |
| **TDZ_ip** | **29.0 (1.3)** | **36.7 (0.4)** |
| **DCX_ip+TDZ_ip** | **28.9 (1.6)** | **36.8 (0.7)** |
| **VAN_ip** | **28.0 (1.4)** | **36.7 (0.6)** |
| **DCX_x1.5** | **26.5 (1.3)** | **38.8 (0.3)** |
| **TDZ_x1.5** | **26.7 (2.1)** | **38.3 (0.2)** |
| **DCX_x.1.5+TDZ_x1.5** | **26.4 (1.2)** | **38.3 (0.5)** |
| **DCX_x4** | **28.3 (1.8)** | **35.3 (0.5)** |
| **TDZ_x4** | **29.3 (0.9)** | **35.2 (1.0)** |
| **DCX_x4+TDZ_x4** | **29.7 (1.4)** | **35.5 (0.5)** |
| **DCX_x1+TDZ_x4** | **26.6 (0.6)** | **37.3 (0.4)** |
